# A real-world study of the effectiveness and safety of low-dose immunotherapy in addition to oral metronomic chemotherapy in recurrent and metastatic head and neck squamous cell carcinoma

**DOI:** 10.1186/s43046-026-00356-9

**Published:** 2026-06-01

**Authors:** Rushabh Kothari, Avinash Khadela, Gaurang Modi, Itesh Khatwani, Palak Bhatt, Vaishnavi Patel, Vraj B. Shah

**Affiliations:** 1 Oncowin Cancer Centre, Ahmedabad, India; 2 L. M. College of Pharmacy, Ahmedabad, India

**Keywords:** Low-dose nivolumab, Immunotherapy, Triple metronomic chemotherapy, Recurrent or metastatic head and neck squamous cell carcinoma (R/M HNSCC)

## Abstract

**Background:**

The standard of care in recurrent/metastatic head and neck squamous cell carcinoma is Pembrolizumab with/without chemotherapy. However, these regimens are minimally affordable and accessible in resource-limited countries. Hence, immunotherapy is combined with oral metronomic chemotherapy in lower doses. Currently, the efficacy and safety of this regimen have been studied in platinum-refractory settings and not beyond them. Thus, we conducted a real-world study on the effectiveness of low-dose immunotherapy and metronomic chemotherapy in recurrent/metastatic head and neck cancer.

**Methodology:**

This was a multicentre, real-world, prospective observational study. Recurrent/metastatic head and neck squamous cell carcinoma patients treated with palliative intent with an Eastern Cooperative Oncology Group performance status of 0–2 were recruited from three oncology centres. Patients received oral metronomic chemotherapy with IV nivolumab 20 mg once every 3 weeks. Patients received treatment until disease progression or unacceptable toxicities. The primary endpoints were Objective response rate, progression-free survival, and Overall survival. The secondary endpoint was safety. Moreover, subgroup analysis was also carried out among platinum sensitivity and the line of treatment.

**Results:**

Overall, 123 patients were recruited (86% males and 35% females). The Objective response rate was 61.7% (95% CI, 53 to 70). The median overall survival and progression-free survival were 15.1 months (95% CI, 12.8–17.4) and 10.4 months (95% CI, 8.0-13.7), respectively. Moreover, the median overall survival was 15.9 (95% CI, 12.7 to 17.5) versus 8.0 months (95% CI, 3.4–12.6) (*p* < 0.01) and the median progression-free survival were 13.6 months (95% CI, 9.9–17.2) versus 5.0 months (95% CI, 2.2–7.8) (*p* < 0.01) for platinum sensitive and resistant subgroups respectively. The median OS were 15.1 months (95% CI, 12.7–17.5) versus 8.0 months (3.4–12.6) (*p* = 0.03), and the median PFS were 11.2 months (95% CI, 6.3–16.0) versus 6.0 months (95% CI, 2.7–9.3) (*p* = 0.07) for first and second-line subgroups, respectively. The most common adverse events were fatigue (63.4%), anemia (52.8%), mucositis (47.9%), and rashes (44.7%).

**Conclusion:**

This study demonstrated that adding low-dose immunotherapy to oral metronomic chemotherapy was effectively applicable and associated with manageable toxicities in a real-world setting for the management of R/M HNSCC. Furthermore, this regimen was associated with favourable survival outcomes and response rates in the platinum-sensitive and first-line treatment subgroups.

**Supplementary Information:**

The online version contains supplementary material available at 10.1186/s43046-026-00356-9.

## Introduction

As per GLOBOCAN 2022, head and neck squamous cell carcinoma (HNSCC) is the 7th most common cancer worldwide, with nearly 660,000 new cases and more than 325,000 deaths annually [1]. Among these, approximately 60% of cases are estimated to be presented at an advanced stage (stage III/IV), and more than 50% of cases still recur due to failure of initial treatment [2]. Despite the advanced stage of the disease, some patients are eligible to undergo surgery, but most patients receive palliative chemotherapy for recurrent/metastatic Head and neck squamous cell carcinoma (R/M HNSCC). Based on the findings of KEYNOTE-048, pembrolizumab with or without chemotherapy is recommended as the first line of treatment for the R/M setting, according to the National Comprehensive Cancer Network (NCCN) and the European Society of Medical Oncology (ESMO) guidelines [3, 4]. However, these regimens have enormous cost implications and, hence, are only accessible to < 5% of the global population and 1–3% of low-middle-income countries (LMIC) [5]. Hence, for this patient population, the EXTREME regimen is considered the first line of treatment in platinum-sensitive HNSCC. Even though this regimen is routinely administered on an outpatient basis in developed countries, it is still poorly tolerated (82% grade ≥ 3 adverse events) and requires hospitalization among LMIC patients to ensure adequate hydration and vigilance over severe toxicities [6]. To overcome these limitations, oral metronomic chemotherapy (OMCT) is administered as a combination of low-dose methotrexate (9 mg/m² weekly), erlotinib (150 mg daily), and celecoxib (200 mg twice daily) over an extended period. Currently, this regimen is rarely utilized in developed countries, and its application is limited to LMICs [7]. Despite higher response rates (42.9%), the duration of response is transitory in these regimens [8].

Immune Checkpoint Inhibitors (ICIs) like nivolumab have a poor response rate in a platinum-refractory setting (13.3%) but demonstrate a sustained duration of response (DOR), leading to a higher OS (7.5 months) [9]. Hence, in the context of these benefits, OMCT is combined with nivolumab. Additionally, a single dose (0.3 mg/kg) of nivolumab is capable of blocking 70% of PD-1 receptors expressed on approximately 20–40% of peripheral T-cells, suggesting its efficacy even at lower doses [10]. These benefits were confirmed in a randomized controlled phase 3 superiority trial (CTRI/2020/11/028953) conducted by Patil et al., which showcased a significant improvement in survival outcomes, tolerability, and quality of life with the use of low-dose nivolumab at 20 mg every 3 weeks with OMCT (TMC-I) compared to OMCT alone (TMC). Since the full dose of nivolumab is financially inaccessible to LMIC, lower doses were used in TMC-I. Remarkably, TMC-I led to an absolute improvement in 1-year OS rates to > 25% and reduced the cost of treatment to 5–9% of the costs of full-dose immunotherapy regimens [11]. Hence, it can be a viable alternative for patients unable to afford full-dose immunotherapy. Therefore, we aim to assess the effectiveness and tolerability of low-dose nivolumab with metronomic chemotherapy in a real-world setting.

## Methodology

### Study design and population included

**Fig. 1:**
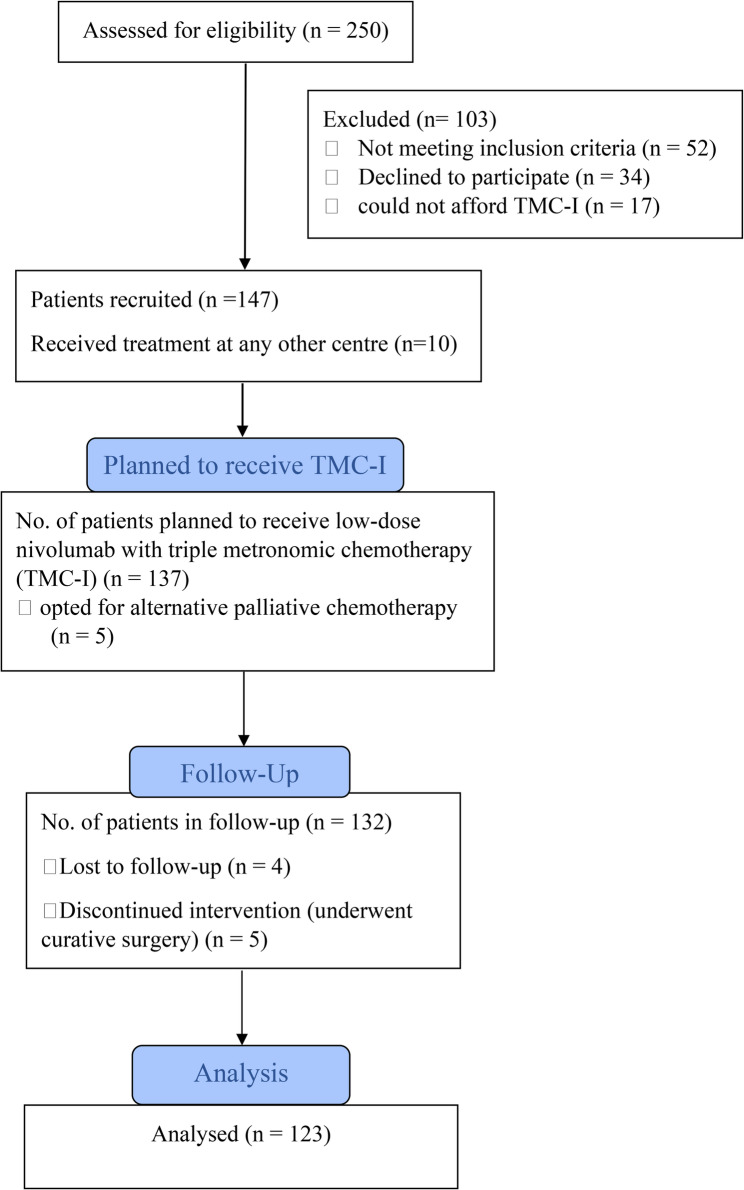
Consort diagram describing the patient selection process as per eligibility criteria

This was a prospective, multicenter, real-world study. The study was approved by the Institutional Review Board (IRB). All patients provided written informed consent before participating in the study. The Recruitment of patients was from August 09, 2022, to June 20, 2024. A total of 250 adult patients were screened, and 123 patients receiving treatment with a palliative intent for HNSCC, with an Eastern Cooperative Oncology Group performance status (ECOG PS) of 0–2, were included in the study. Pregnant and lactating women, patients with autoimmune diseases, patients receiving immunosuppressants, and those with uncontrolled comorbidities were excluded (Fig. 1).

### Treatment characteristics

A detailed history and clinical examination were performed on all patients. Blood investigations, including a complete blood count, renal function, and liver function, were performed at baseline. Axial imaging with either contrast-enhanced computed tomography or magnetic resonance imaging was performed. Patients received TMC-I, which consisted of celecoxib 200 mg twice daily, methotrexate 9 mg/m^2^ orally once a week, and erlotinib 150 mg once daily orally. Additionally, patients also received 20 mg nivolumab intravenously once every 3 weeks in 100 mL of normal saline over 60 min. The commercially available 40 mg nivolumab vial was shared among the patients. The patients underwent axial scans every 2 months for assessment of response, and were independently reviewed by a team of radiologists and medical oncologists. The adverse events were examined according to the Common Terminology Criteria for Adverse Events version 4.03 (CTCAE 4.03). The patients continued to receive the treatment until the emergence of disease progression or intolerable adverse events.

### Endpoints

The primary endpoints of the study were Overall survival (OS), Progression-free survival (PFS), Objective response rate (ORR), and the Duration of Response (DOR). The OS was defined as the time from the date of initiation of treatment until death. Patients who were alive were censored at the time that they were last known to be alive. The PFS was calculated from the date of initiation of treatment to the date of progression or death, whichever was earlier. The DOR was calculated from the date of first documentation of objective tumor response (complete or partial) until disease progression or death. The OS, PFS, and DOR were analysed using the Kaplan-Meier survival analysis, and the difference between the survival curves in each subgroup was analysed using the log-rank test. The ORR was calculated as a percentage of patients having a complete response (CR) and partial response (PR) as the best response, which was assessed following the Response Evaluation Criteria in Solid Tumors (RECIST) version 1.1 criteria. The secondary endpoint included safety.

### Statistical analysis

The statistical analysis was performed by using IBM SPSS version 22. Descriptive statistics were performed. Ordinal and nominal variables were compared using the chi-square and Fisher’s test. The Kaplan-Meier method was used to estimate the median OS and PFS. The log-rank test was used to compare OS and PFS, respectively, in subgroup analysis between platinum-sensitive (patients who initially respond to platinum-based chemotherapy and then recur after at least six months following treatment) and platinum-resistant subgroups (patients who initially respond to platinum-based chemotherapy but then progress or recur within six months following treatment) and first-line/second-line treatment groups. Factors affecting OS and PFS were also sought via the COX regression model by calculating the hazard ratio (HR) with its 95% CI.

## Results

### Baseline characteristics

Overall, 123 Patients who received TMC-I for palliative intent were recruited from three oncology centres located in Ahmedabad, Gujarat, India. The recruitment occurred from August 09, 2022, to June 20, 2024. The baseline characteristics of patients were analysed, and a detailed description is provided in Table 1. In baseline characteristics, Male patients (86%) demonstrated a higher proportion of patients than females (14%). A majority of patients (67%) were between 40 and 60 years of age. The buccal mucosa subsite (OCM) was the most prominent (59.4%) subsite for oral cavity cancer, and 95.1% of patients had an ECOG performance status score of 1. Moreover, 41% of patients presented with a recurrent disease, 29% patients presented as upfront metastatic disease, and 30% patients progressed while receiving treatment with curative intent.

**Table 1:** Demographic details and disease-related characteristics of R/M HNSCC patients

Demographic parameter	Number of patients (*n* = 123) (%)
Gender	
Male	106 (86)
Female	17 (14)
Age groups, years	
30–40	25 (20)
40–50	43 (35)
50–60	40 (32)
> 60	15 (13)
Subsites	
OCM	73 (59.4)
OCT	50 (40.6)
ECOG Performance status	
1	117 (95.1)
2	6 (4.9)
Recurrent/Metastatic status	
Local or regional recurrence without distant metastasis	50 (41)
Upfront metastatic	36 (29)
Progressed locally and regionally while receiving treatment for early-stage disease.	31 (25)
Progressed while receiving alternative medicine (ayurvedic, homeopathic, unnani, etc.)	6 (5)
Previous treatment	
Surgery alone	6 (5)
CT alone	26 (21)
Surgery + RT	15 (12)
Surgery + CT + RT	28 (23)
CT + RT	6 (5)
Treatment naive	36 (29)
Alternative medicine	6 (5)

*Abbreviations*: *CT* Chemotherapy, *RT* Radiotherapy, *OCM* Oral Cavity Buccal Mucosa, *OCT* Oral Cavity Tongue , *ECOG* Eastern Cooperative Oncology Group

### Sub-group analysis of baseline characteristics

Sub-group analysis was carried out by dividing the groups as per the following criteria: (1) Platinum sensitivity (platinum sensitive and platinum resistant), and (2) Line of treatment (first and second line). Among 123 patients, 82 patients were platinum-sensitive, and 41 patients were in the platinum-resistant subgroup. Similarly, 89 patients were in the first-line subgroup, and 34 were in the second-line subgroup. Tables 2 and 3 summarize the baseline characteristics for the respective subgroups.

**Table 2:** Demographic details and disease-related characteristics of 123 R/M-HNSCC patients with platinum-sensitive and platinum-resistant disease

Demographic parameter	Number of patients (%) in platinum-sensitive subgroup (*n* = 82)	Number of patients (%) in platinum-resistant sub-group (*n* = 41)	*p*-value
Gender			
Male	68(55.2)	38(30.9)	0.173
Female	14 (11.4)	3(2.5)	
Age groups, (years)			
30–40	16(13)	9 (7.3)	0.784
40–50	25(20.3)	18 (14.6)	
50–60	30 (24.3)	10 (8.3)	
> 60	11(8.9)	4 (3.2)	
Subsites			
OCM	46 (37.4)	27 (21.9)	0.335
OCT	36 (29.3)	14 (11.4)	
ECOG Performance status			
1	78 (63.4)	39 (31.7)	0.683
2	4 (3.3)	2 (1.6)	

Abbreviations: *OCM* Oral Cavity Buccal Mucosa, *OCT* Oral Cavity Tongue, *ECOG* Eastern Cooperative Oncology Group, *R/M HNSCC* Recurrent or metastatic head and neck squamous cell carcinoma

**Table 3:** Demographic details and disease-related characteristics of 123 R/M-HNSCC patients treated in first line or in second line

Demographic parameter	Number of patients (%) in First line TMC-I regimen sub-group (*n* = 89)	Number of patients (%) in Second line TMC-I regimen sub-group (*n* = 34)	*p*-value
Gender			
Male	76 (61.8)	30 (24.4)	0.467
Female	13 (10.6)	4 (3.2)	
Age groups (years)			
30–40	20 (16.3)	5 (4)	0.386
40–50	26 (21.1)	17 (13.8)	
50–60	32 (26.1)	8 (6.6)	
> 60	11 (8.9)	4 (3.2)	
Subsites			
OCM	54 (43.9)	19 (15.4)	0.684
OCT	35 (28.5)	15 (12.2)	
ECOG Performance status			
1	84 (68.4)	33 (26.8)	0.464
2	5 (4)	1 (0.8)	

*Abbreviations*: *OCM* Oral Cavity Buccal Mucosa, *OCT* Oral Cavity Tongue, *ECOG* Eastern Cooperative Oncology Group, *R/M HNSCC* Recurrent or metastatic head and neck squamous cell carcinoma

### Objective response rate and duration of response

**Fig. 2:**
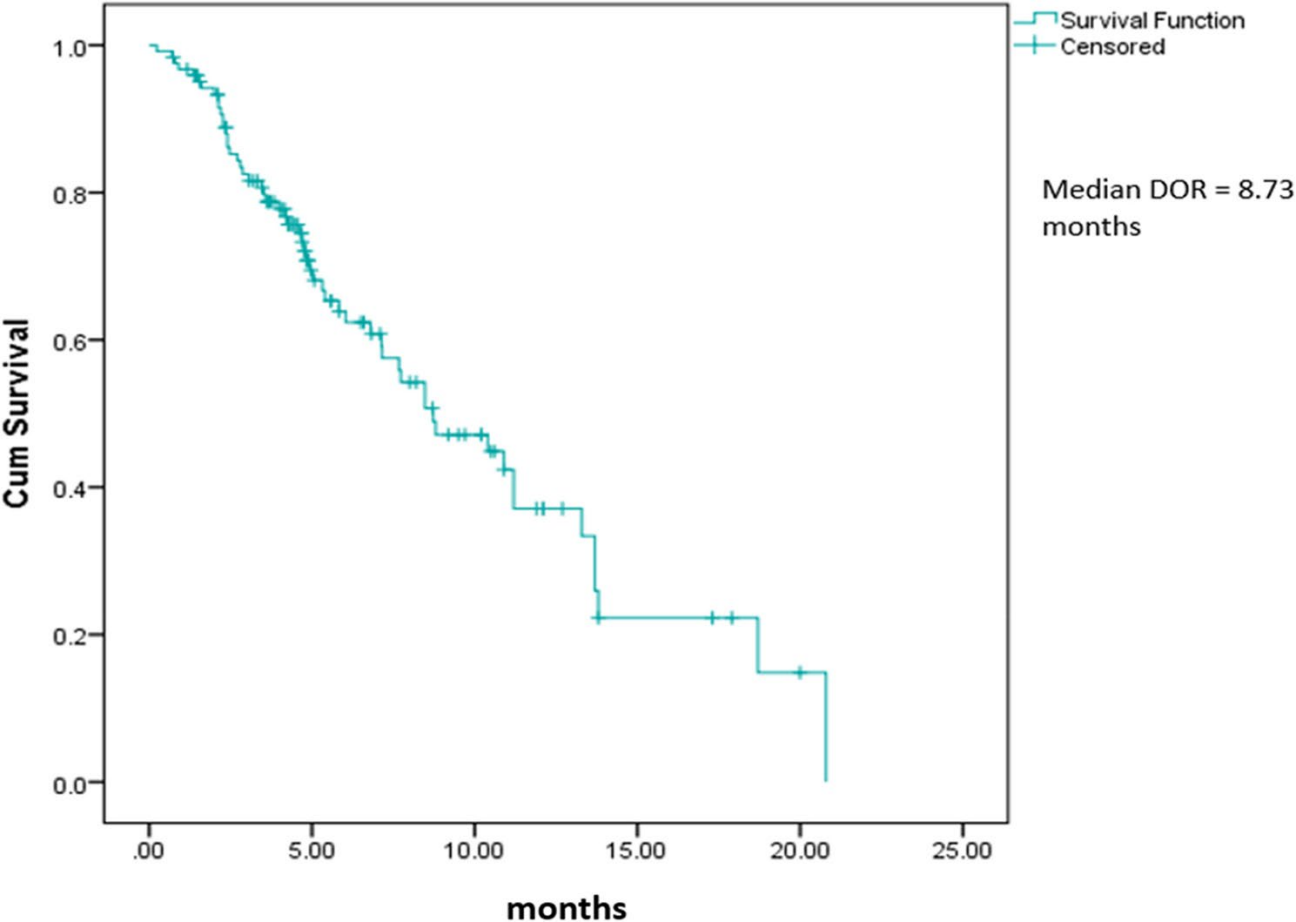
Kaplan-Meier curves for duration of response (DOR)

**Fig. 3:**
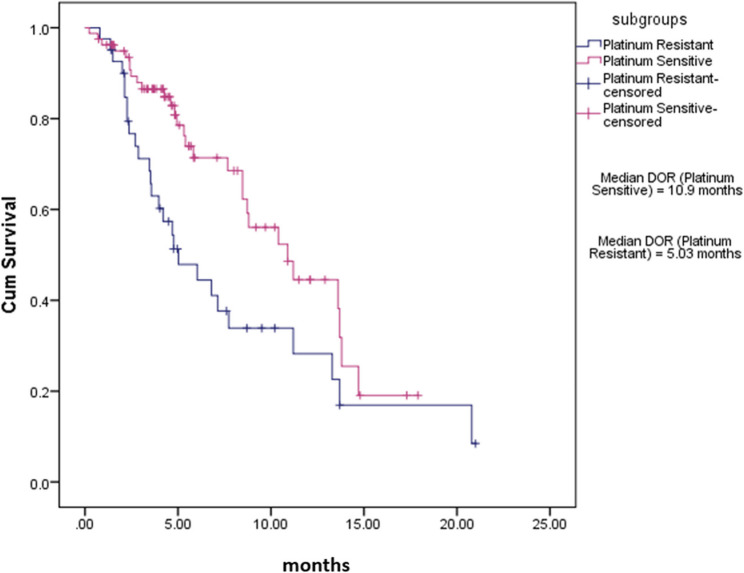
Kaplan Meier curves for duration of response (DOR) for platinum sensitive and platinum resistant subgroups

**Fig. 4:**
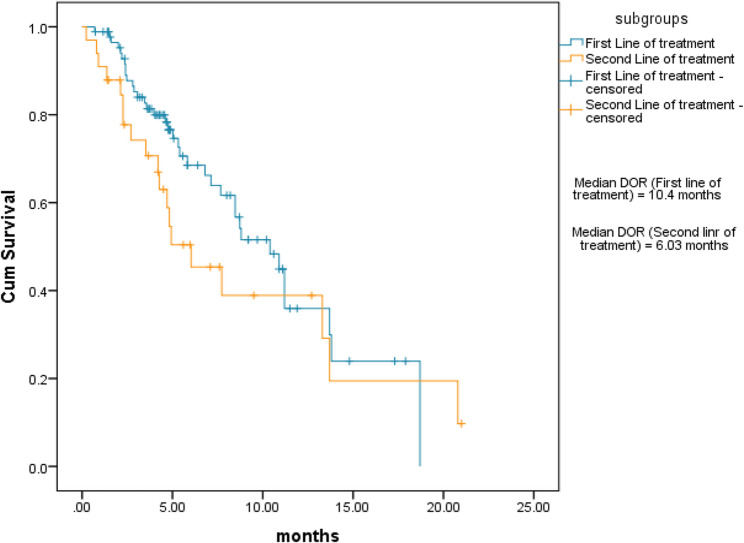
Kaplan Meier curves for duration of response (DOR) among first-line and second-line treatment subgroups

The overall objective response rate (ORR) was 61.7% (95% CI, 53 to 70). Additionally, the ORR was 67.0% (95% CI, 56.9 to 77.2) for platinum-sensitive and 48.78% (95% CI, 33.4 to 64) for platinum-resistant subgroups, respectively. The ORR for the first-line and second-line treatment sub-groups was 65.1% (95% CI, 55 to 75) and 52.9% (95% CI, 36.1 to 69.7), respectively. The overall median DOR was 8.73 months (95% CI, 5.89 to 11.86). The median DOR curve is provided in Fig. 2. Furthermore, the median DOR for the platinum-sensitive subgroup was 10.90 months (95% CI, 7.48 to 14.31), while that of the platinum-resistant subgroup was 5.03 months (95% CI, 2.68 to 7.31). The log-rank test demonstrated a statistically significant difference (*p* = 0.01) in the median DOR among this subgroup. Among the patients receiving TMC-I as the first line of treatment, the median DOR was 10.40 months (95% CI, 7.44 to 13.35) versus 6.02 months (95% CI, 3.03 to 8.99) for patients receiving this regimen as the second line of treatment. However, the median DOR curves for this subgroup did not demonstrate statistical significance (*p* = 0.28). The median DOR curves as per subgroup analysis for platinum-sensitive subgroups and line of treatment-based subgroups are shown in Figs. 3 and 4, respectively.

### Overall survival

**Fig. 5:**
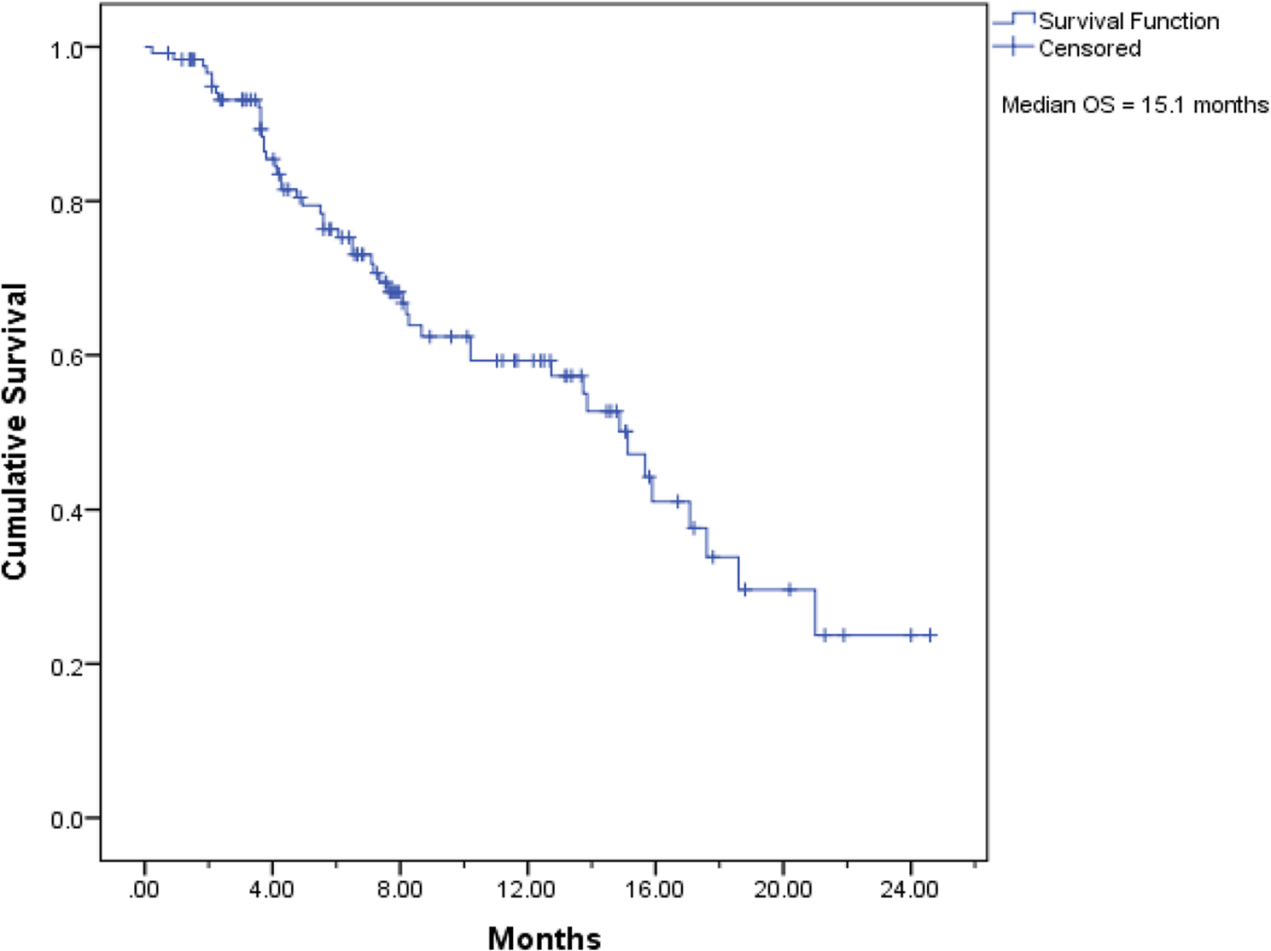
Kaplan-Meier curve for median overall survival (OS)

**Fig. 6:**
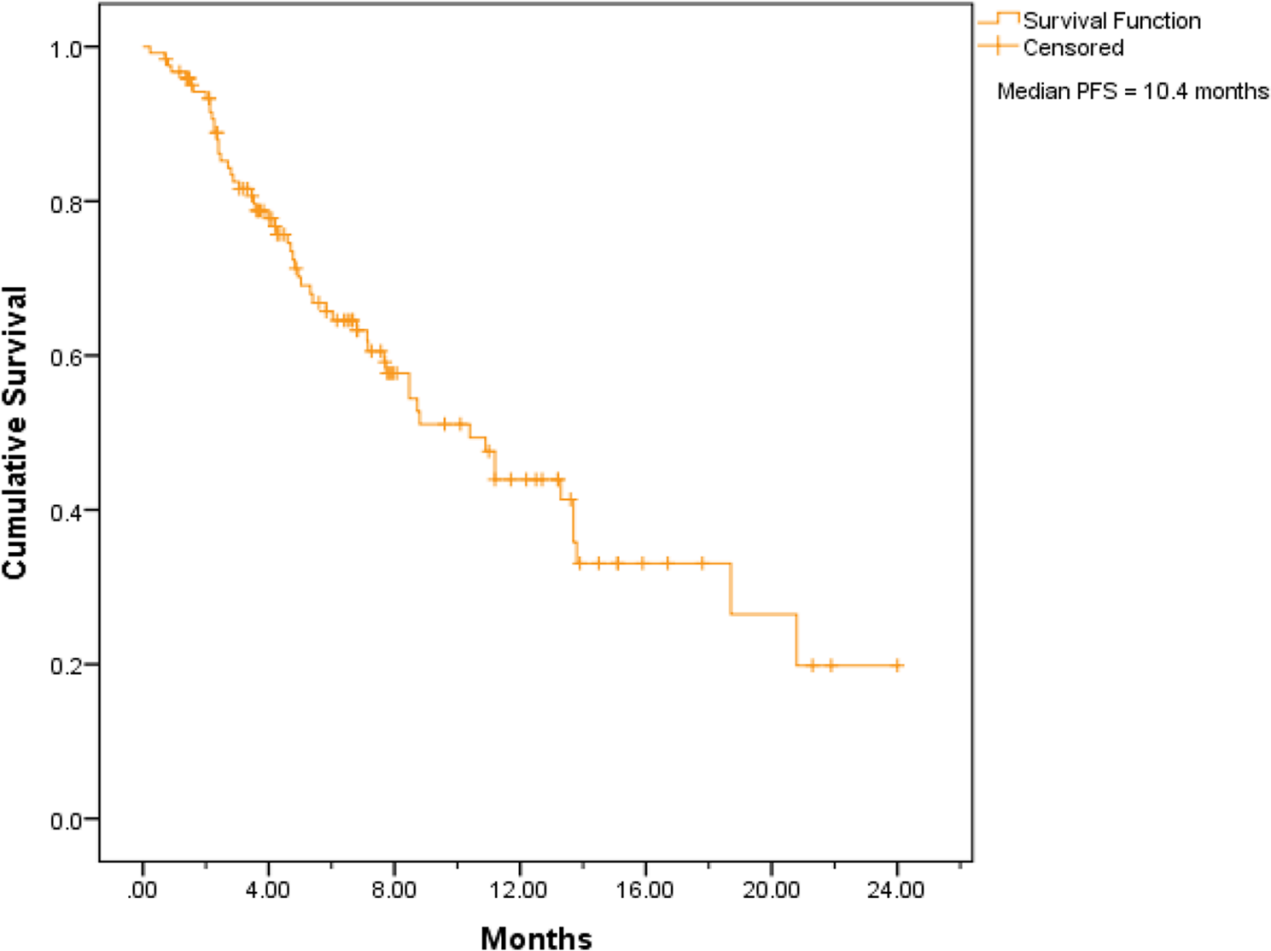
Kaplan-Meier curves for median progression-free survival (PFS)

**Fig. 7:**
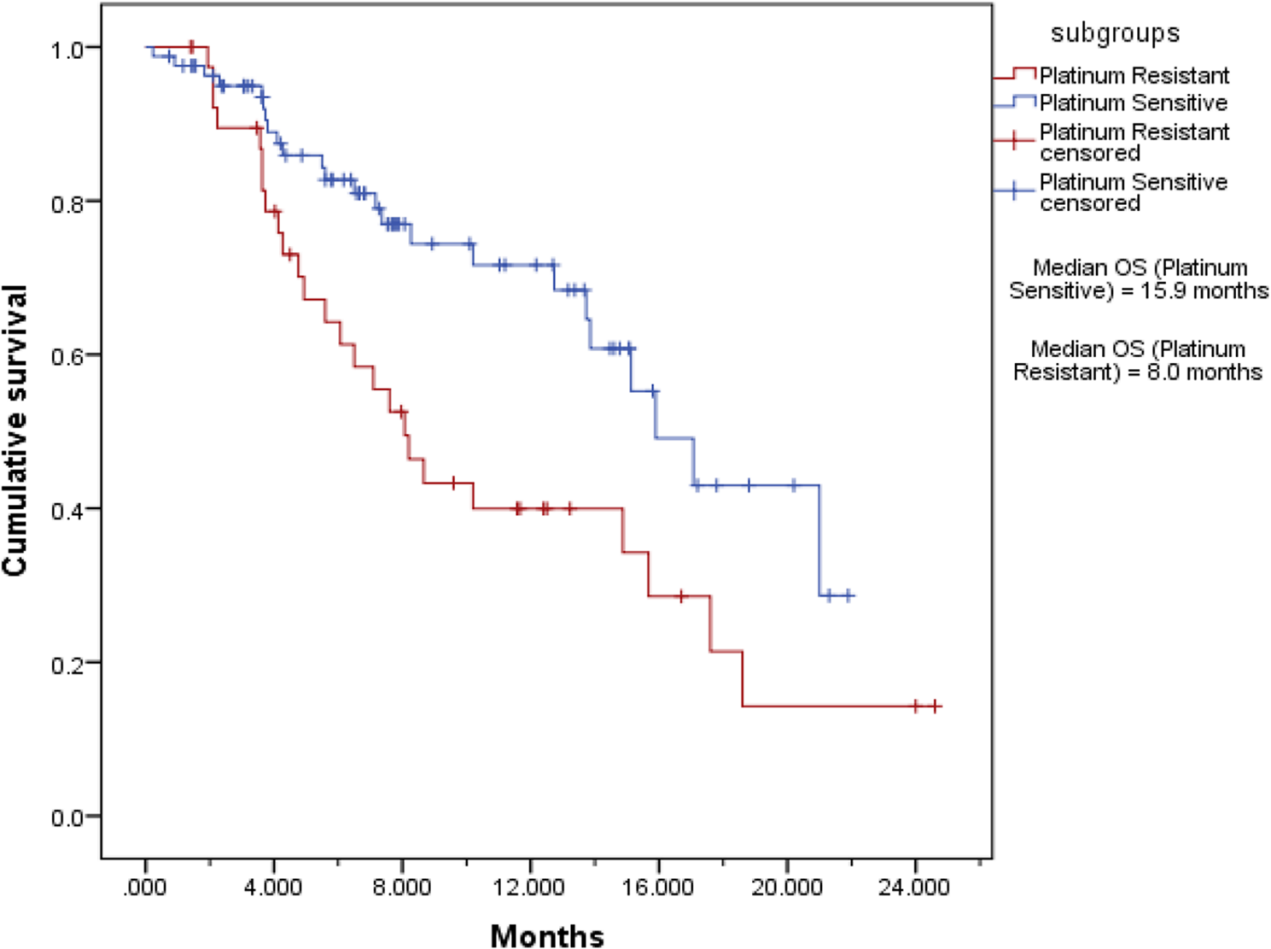
Kaplan-Meier curves for overall survival (OS) for platinum sensitive and platinum resistant subgroups

**Fig. 8:**
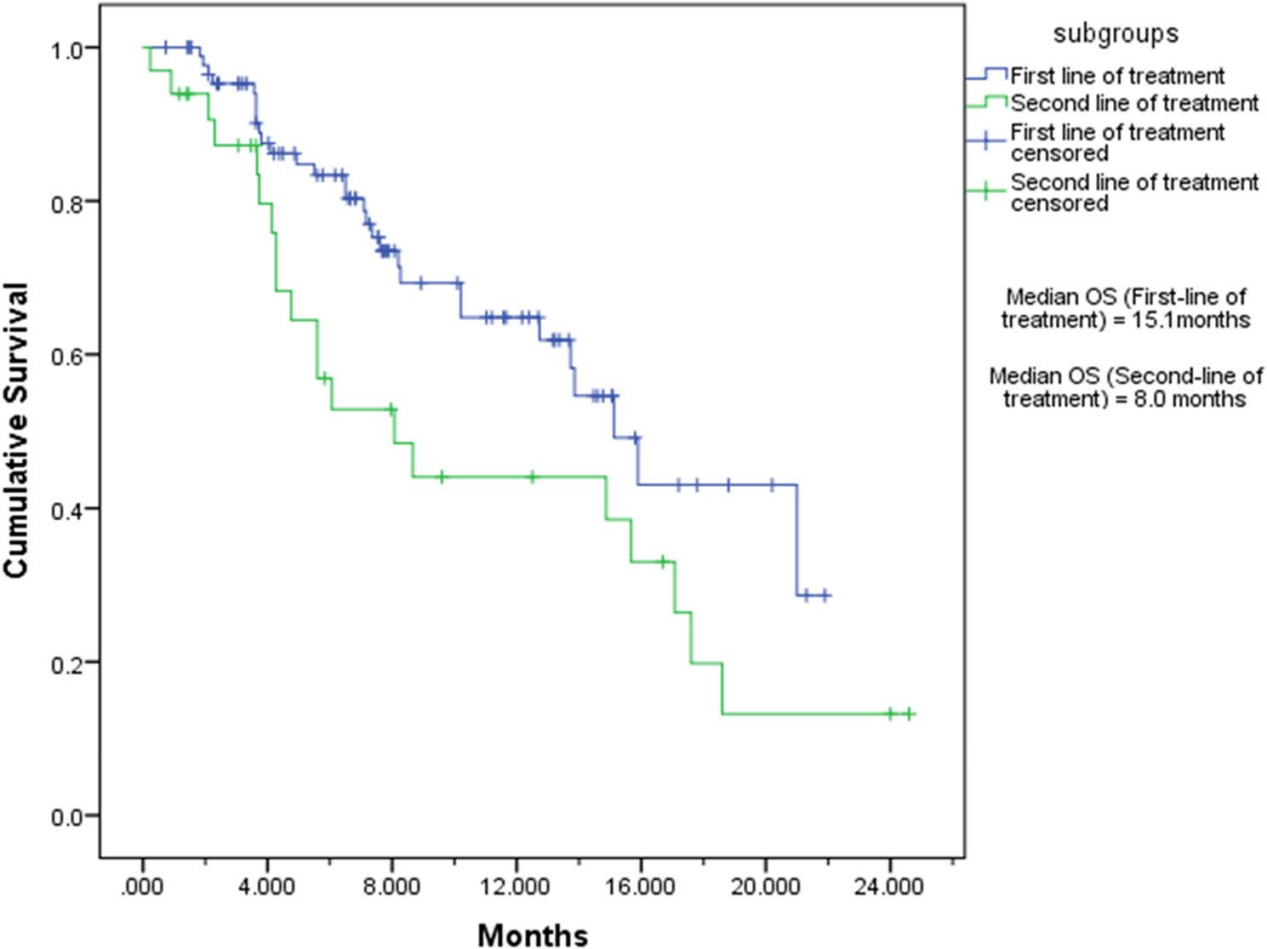
Kaplan-Meier curves for overall survival (OS) among first-line and second-line treatment subgroups

The 1-year OS was 57.3% (95% CI, 51.8 to 62.8), and the median OS was 15.1 months (95% CI, 12.8 to 17.4). The median OS curve is provided in Fig. 5. Additionally, the median OS was 15.9 (95% CI, 12.8 to 18.9) and 8 months (95% CI, 5.9 to 10.1) for platinum-sensitive and platinum-resistant subgroups, respectively, and the log-rank test indicates a statistically significant difference amongst overall survival curves (*p* < 0.01). There were 49 deaths in total, with 24 deaths in the platinum-sensitive and 25 deaths in the platinum-resistant subgroups. For the patients receiving TMC-I as First and Second lines of treatment, the median OS was 15.1 months (95% CI, 12.7 to 17.5) and 8 months (95% CI, 3.4 to 12.6), respectively. The log-rank test indicated a statistically significant difference among OS curves (*p* = 0.03). Additionally, 29 deaths occurred in patients receiving TMC-I as a first-line line, and 20 deaths occurred in patients receiving TMC-I as a second-line treatment. The clinical factors, such as age, subsite, and performance status, did not affect the OS of patients. However, gender significantly impacted the OS, and the male gender was associated with poorer survival outcomes. The multivariate analysis for OS is shown in Table 1 (See Table 1 of the ESM), and OS curves as per subgroup analysis for platinum-sensitive subgroups and line of treatment-based subgroups are shown in Figs. 6, 7 and 8.

### Progression-free survival

**Fig. 9:**
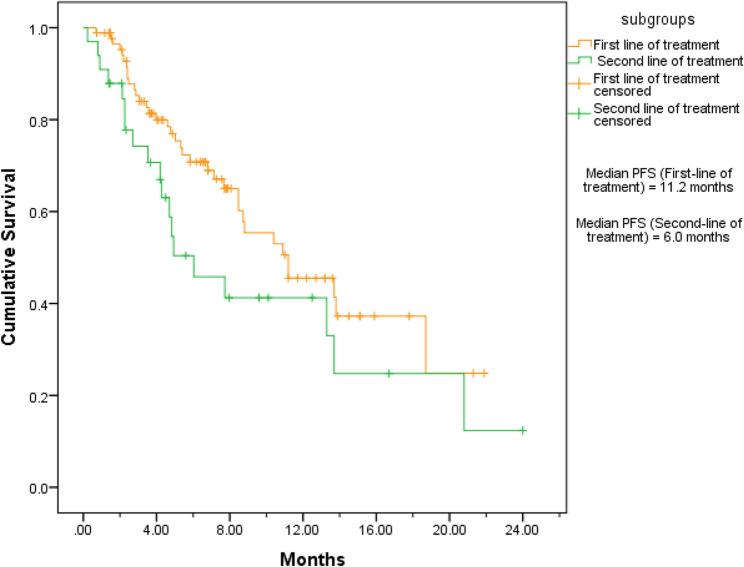
Kaplan Meier curves for progression-free survival (PFS) among first-line and second-line treatment subgroups

**Fig. 10:**
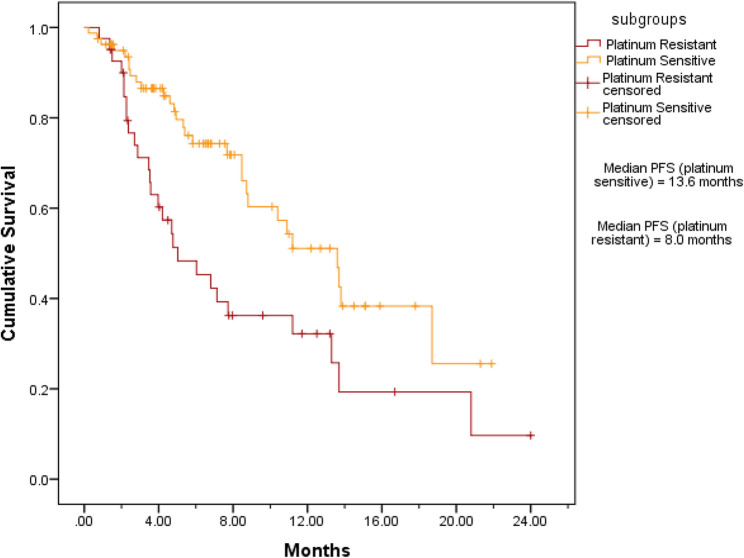
Kaplan Meier curves for progression-free survival (PFS) for platinum sensitive and platinum resistant subgroups

The 1-year PFS was 41.3% (95% CI, 35.3 to 47.3), and the median PFS was 10.4 months (95% CI, 8.0 to 13.7). The median PFS curve is shown in Fig. 6. Additionally, the median PFS was 13.6 months (95% CI, 9.9 to 17.2) and 5 months (95% CI, 2.2 to 7.8) for platinum-sensitive and platinum-resistant subgroups, respectively, and the log-rank test indicated a statistically significant difference amongst PFS curves (*p* < 0.01). There were 56 progression events in total, 29 progression events in platinum-sensitive patients, and 27 progression events in platinum-resistant patients. Among the other subgroups, for the patients receiving TMC-I as first and second lines of treatment, the median PFS was 11.2 months (95% CI, 6.3 to 16.0) and 6.03 months (95% CI, 2.7 to 9.3), respectively. However, the log-rank test does not indicate a statistically significant difference among PFS curves (*p* < 0.07). Additionally, there were 36 progressions in the first-line and 19 progressions in the second-line treatment subgroups, respectively. The clinical factors, such as age, gender, and performance status, did not significantly impact the PFS. The multivariate analysis for PFS is shown in Table 2 (See Table 2 of the ESM), and PFS curves as per subgroup analysis for line of treatment-based and platinum-sensitive subgroups are shown in Figs. 9 and 10.

### Adverse events

The rate of grade 3 or above adverse events occurred in 43% of patients (*n* = 56). The most common adverse events were fatigue (63.4%), anemia (52.8%), neutropenia (26.8%), thrombocytopenia (20.3%), mucositis (47.9%), rashes (43.3%), and diarrhea (39.0%). Deaths due to treatment-related adverse events (TRAE) were not observed in any patient. A detailed list of all the TRAEs is provided in Table 4.

**Table 4:** List of adverse events observed in 123 R/M HNSCC patients

Adverse events	Any grade Number of patients (%)	Grade 3 or above Number of patients (%)
Fatigue	78 (63.4%)	12 (9.8%)
Anemia	65 (52.8%)	7 (5.7%)
Neutropenia	33 (26.8%)	7 (5.7%)
Thrombocytopenia	25 (20.3%)	5 (4.1%)
Mucositis	59(47.9%)	8 (6.5%)
Rashes	55 (44.7%)	9 (7.3%)
Diarrhea	48 (39.0%)	1 (0.8%)
Hypothyroidism	7 (5.7%)	-
Hyponatremia	39 (31.7%)	-
Fever	3 (2.4%)	1 (0.8%)
AST/ALP rise	47 (38.2%)	-
Bleeding	18 (14.6%)	3 (2.4%)
Rise in serum creatinine	9 (7.3%)	-
Hyperkalemia	28 (22.7%)	2 (1.6%)

*Abbreviations*: *AST* Aspartate Aminotransferase, *ALT* Alanine Aminotransferase

## Discussion

The recent advent of immunotherapy has brought about a paradigm shift in the treatment of R/M HNSCC [12]. However, due to limited access and higher costs, patients in low-middle-income countries (LMIC) like India are unable to afford these regimens. Hence, the treatment options for this population are limited to platinum or 5-FU-based chemotherapy with/without cetuximab for platinum-sensitive and OMCT for platinum-refractory patients [2, 6, 13]. These regimens are associated with higher toxicities, poor response rates, and survival outcomes in the R/M setting [14]. To address these limitations, the addition of low-dose immunotherapy to OMCT in a platinum-refractory setting was explored by V.M. Patil et al. in a randomized controlled phase 3 superiority trial (CTRI/2020/11/028953) [11]. However, clinical trials only represent a minority of the real-world population because of stringent eligibility criteria. For instance, in real-world settings, TMC-I is occasionally utilized as a first-line treatment for R/M HNSCC longer than six months after platinum-containing chemotherapy, i.e., platinum-sensitive patients for better prognostic outcomes and quality of life. Moreover, a handful of patients in a real-world setting also present with non-oral cavity cancers and poorer ECOG PS (2–3), which may affect the overall prognosis of these patients. Hence, we conducted a prospective observational study to assess the real-world efficacy and safety of low-dose nivolumab along with OMCT to define its utility in the treatment paradigm of R/M HNSCC.

To the best of our knowledge, this is the first real-world study of the addition of low-dose immunotherapy along with OMCT in the Indian population. The patients receiving TMC-I demonstrated a 1-year OS of 57.3% and 1-year PFS of 41.3%. Additionally, the median OS, PFS, and DOR in this study were 15.1, 10.4, and 8.73 months, respectively. These survival rates were substantially higher compared to studies conducted by V.M. Patil et al. and S.K. Swain et al. that reported a 1-year OS of 43.4% and 37.5%, respectively. The median OS was 10 months, and the median PFS was 4 to 7 months, respectively, in both of these studies. However, a similar median DOR was reported in our study (8.73 months) and the randomized phase 3 trial conducted by V.M. Patil et al. (8.70 months) [15].

The ORR in this study reached up to 61.7%, with 4.8% CR, 9.7% Stable Disease (SD), and 56.9% patients having partial response (PR). These findings were consistent with studies conducted by V.M. Patil et al. and S.K. Swain et al. that reported an ORR of 59.2% and 56%, respectively. The ORR, median PFS, and median OS of these studies and our study are summarized in Table 5. The rate of grade 3 or higher adverse events was 44.7%, which was consistent with both the studies that reported 43% to 46% occurrence of grade 3 or higher adverse events [11, 16, 17]. This study demonstrated a substantially higher survival rate, median OS, and PFS compared to studies conducted by V.M. Patil et al. and S.K. Swain et al. The primary underlying factor contributing to this was that in this study, a higher number of patients were platinum-sensitive and received TMC-I as first-line treatment. Similarly, these benefits are also reflected across a Phase 4 superiority trial conducted by Khadela et al., which reports a median OS of 15.1 months and a median PFS of 11.4 months [18]. Moreover, the OS, PFS, and DOR curves demonstrated that TMC-I has better prognostic outcomes in a platinum-sensitive setting. Hence, corroborating that the improved survival outcomes are attributable to the higher response rates and duration of responses obtained in these subgroups, and thus account for a better prognosis.

**Table 5:** Results of previous clinical studies

Study title	ORR	Median PFS	Median OS
V.M. Patil et al.	59.2%	6.6 months	10.1 months
S.K. Swain et al.	56.0%	7 months	10.2 months
This study	61.7%	10.4 months	15.1 months

*Abbreviations:* *ORR *Objective Response Rate,* PFS *Progression Free Survival,*OS *Overall Survival

Historically, platinum resistance is associated with poor response to subsequent chemotherapy and, hence, a worse prognosis in many cancers, including HNSCC [19]. Moreover, numerous real-world studies have also reported better survival outcomes in platinum-sensitive patients with the use of the EXTREME regimen in R/M HNSCC [20, 21]. Hori R. et al. conducted a real-world study of full-dose nivolumab, which identified that platinum-resistant carcinoma is an independent negative predictor of both PFS and OS. The patients with platinum-resistant carcinoma had a substantially lower 1-year PFS rate than those with platinum-sensitive carcinoma, i.e., 18.0% versus 43.6%. The 1-year OS rates were 44.3% and 84.0% for platinum-resistant and platinum-sensitive carcinoma, respectively. This suggests that rapid progression following platinum-containing chemotherapy has been associated with poor prognosis, even with the use of ICIs [22]. Another study conducted by Isaku Okamoto et al. reported a median PFS and OS of 9.6 and 17.4 months, respectively, with the use of full-dose nivolumab in a platinum-sensitive setting [19]. This further adds to the benefits of early exposure to immunotherapy in R/M HNSCC. Furthermore, these findings were confirmed in a randomized phase 3 KEYNOTE-048 trial with the use of pembrolizumab with or without chemotherapy, which led to its approval as a first-line treatment in the R/M setting [3]. However, the underlying mechanism for this improved response in platinum-sensitive patients is still unknown. The benefits of full-dose immunotherapy in a platinum-sensitive setting are verified via several studies, but that of adding low-dose immunotherapy to OMCT needs further evaluation in large-scale randomized trials.

Apart from a platinum resistance, the multivariate analysis in this study for clinical factors demonstrated that gender positively impacted the OS. The female gender has a favourable OS compared to the male. These findings were consistent with other studies conducted by Angela. L. Mazul et al. and Bhargan Ram et al. [23, 24]. This study also explored the effectiveness of TMC-I as first and second lines of treatment. Among 123 patients, 89 (72.35%) received TMC-I as first line, and 34 (27.64%) received it as second line of treatment. The median OS and PFS for the first-line subgroup were 15.13 and 11.20 months, respectively, while those of the second-line subgroup were 8.06 and 6.03 months, respectively. The ORR in the first line and second line of treatment were 65.17% and 52.94%, respectively. This suggests that TMC-I in the first line of treatment improved the OS as a result of improvement in PFS and ORR. A disease progression secondary to initial lines of treatment results in micro-metastasis of the disease, which eventually reduces the effectiveness of the treatment [25]. Moreover, previous lines of chemotherapy may also reduce the performance status and quality of life of the patient due to increased toxicities [26]. This may reduce the tolerance of the patient to the next lines of treatment and ultimately lead to progression [27]. Despite a substantial clinical and prognostic distinction between the subgroups, the PFS curves did not demonstrate difference in survival outcomes. These findings could be attributed to a smaller sample size of the second-line subgroup, which was only composed of 27% of patients.

The limitation of our study is that this study was only performed on patients having oral cavity cancers. The HPV positive and non-oral cavity cancer patients were not included. Apart from this, several outcomes remain unexplored, like quality of life and patient compliance. This study also does not include a comparative group, and the effectiveness of low-dose nivolumab with OMCT as compared to full-dose immunotherapy has not been carried out in this study. Moreover, this study also lacks the Programmed Death Ligand-1 (PDL-1) Combined Positive Score (CPS) in the study participants, which may confound the interpretation of survival curves among the sub-groups as patients having greater expression of PDL-1 shows better response to immunotherapy. 

## Conclusion

In conclusion, this real-world study of combining low-dose immunotherapy with oral metronomic chemotherapy demonstrated that this regimen was effectively applicable and well tolerated in a real-world setting in recurrent/metastatic head and neck squamous cell carcinoma patients receiving treatment with palliative intent. The findings obtained from this study corroborate the use of low-dose immunotherapy along with oral metronomic chemotherapy in a platinum-sensitive setting as a first-line treatment. This is especially attributed to the patients who are ineligible to obtain the standard of care regimens recommended by NCCN and ESMO, either because of toxicities or cost implications. However, further evaluation of this regimen in a larger population is required, and additional clinical studies comparing the efficacy of low-dose immunotherapy with full-dose immunotherapy are also necessary. This is to determine whether low-dose immunotherapy, along with metronomic chemotherapy, can serve as an alternative standard of care for patients who cannot afford the cost of full-dose immunotherapy-based regimens.

## Supplementary Information

Supplementary Material 1.

## Data Availability

The Datasets used or analysed during current study will be available upon reasonable request from corresponding author.

## Abbreviations

R/M HNSCC: Recurrent or metastatic head and neck squamous cell carcinoma
TMC-I: Low-dose nivolumab with oral metronomic chemotherapy
LMIC: Low and middle-income countries
ORR: Objective response rate
PFS: Progression-free survival
OS: Overall survival
ECOG PS: Eastern Cooperative Oncology Group performance status
OCM: Oral cavity buccal mucosa
OCT: Oral cavity tongue
CTCAE 4.03: Common Terminology Criteria for Adverse Events
RECIST 1.1: Response Evaluation Criteria in Solid Tumors 1.1
AST: Alanine Aminotransferase
ALT: Aspartate Aminotransferase
