# Supplementary material for: A real-world study of the effectiveness and safety of low-dose immunotherapy in addition to oral metronomic chemotherapy in recurrent and metastatic head and neck squamous cell carcinoma
Source: J Egypt Natl Canc Inst. 2026 Jun 1;38:18. doi: 10.1186/s43046-026-00356-9 (PMC13313305; doi:10.1186/s43046-026-00356-9)
Supplement: Supplementary file 1 — Supplementary Material 1. [file 43046_2026_356_MOESM1_ESM.docx]

**SUPPLEMENTARY APPENDIX**

| **Table of contents** | |
| --- | --- |
| **Table** | **Page no** |
| **Table 1.** Multivariate analysis for factors impacting Overall survival (OS) | 2 |
| **Table 2.** Multivariate analysis for factors impacting Progression-free survival (PFS) | 2 |
| **Table 3:** Number of patients at risk (censored) during specific time intervals (months) for median overall survival (OS) | 3 |
| **Table 4**. Number of patients at risk (censored) during specific time intervals (months) for median progression-free survival (PFS) | 3 |
| **Tables 5 & 6.** Number of patients at risk (censored) during specific time intervals (months) for median overall survival (OS) among platinum-sensitive and Line of treatment subgroups. | 3 |
| **Tables 6 &7.** Number of patients at risk (censored) during specific time intervals (months) for median progression-free survival (PFS) among platinum-sensitive and Line of treatment subgroups. | 4 |

| **Table 1.** Multivariate analysis for factors impacting Overall survival (OS) | | | |
| --- | --- | --- | --- |
| **Factors** | **Hazard Ratio (95% CI)** | **P-value** | **Reference subgroup** |
| **Gender (Male)** | 3.25 (1.00-10.54) | 0.05 | Female |
| **Age (<60 years)** | 1.202 (0.720-2.005) | 0.481 | Age (>60 years) |
| **Subsite (OCM)** | 1.489 (0.8-2.761) | 0.207 | OCT |
| **ECOG PS 1** | 1.594 (0.78-3.25) | 0.201 | ECOG PS 2 |
| **Abbreviations:** Oral Cavity Buccal Mucosa (OCM), Oral Cavity Tongue (OCT), Overall survival (OS), Eastern Cooperative Oncology Group Performance Status (ECOG PS) | | | |

| **Table 2.** Multivariate analysis for factors impacting Progression-free survival (PFS) | | | |
| --- | --- | --- | --- |
| **Factors** | **Hazard Ratio (95% CI)** | **P-value** | **Reference subgroup** |
| **Gender (Male)** | 1.385(0.591-3.246) | 0.454 | Female |
| **Age (<60 years)** | 1.139(0.718-1.806) | 0.518 | Age (>60years) |
| **Subsite (OCM)** | 1.397(0.79-2.45) | 0.244 | OCT |
| **ECOG PS 1** | 1.026(0.615-1.713) | 0.921 | ECOG PS 2 |
| **Abbreviations:** Oral Cavity Buccal Mucosa (OCM), Oral Cavity Tongue (OCT), Progression-free survival (PFS), Eastern Cooperative Oncology Group Performance Status (ECOG PS) | | | |

| **Table 3:** Number of patients at risk (censored) during specific time intervals (months) for median overall survival (OS) | | | | | |
| --- | --- | --- | --- | --- | --- |
| Number of patients at risk | 123 | 70 | 33 | 8 | 1 |
| Months | 0 | 6 | 12 | 18 | 24 |

| **Table 4**. Number of patients at risk (censored) during specific time intervals (months) for median progression-free survival (PFS) | | | | | |
| --- | --- | --- | --- | --- | --- |
| Number of patients at risk | 123 | 56 | 21 | 5 | 0 |
| Months | 0 | 6 | 12 | 18 | 24 |

| **Table 5:** Number of patients at risk (censored) during specific time intervals (months) for median overall survival (OS) among platinum-sensitive subgroups. | | | | | |
| --- | --- | --- | --- | --- | --- |
| Number of patients at risk (Platinum sensitive) | 81 | 48 | 23 | 4 | 0 |
| Number of patients at risk (Platinum resistant) | 39 | 21 | 9 | 2 | 1 |
| Months | 0 | 6 | 12 | 18 | 24 |

| **Table 6:** Number of patients at risk (censored) during specific time intervals (months) for median overall survival (OS) among Line of treatment subgroups. | | | | | |
| --- | --- | --- | --- | --- | --- |
| Number of patients at risk (First-line) | 88 | 56 | 24 | 4 | 0 |
| Number of patients at risk (Second line) | 32, | 13 | 8 | 2 | 0 |
| Months | 0 | 6 | 12 | 18 | 24 |

| **Table 7:** Number of patients at risk (censored) during specific time intervals (months) for median progression-free survival (PFS) among platinum-sensitive subgroups. | | | | | |
| --- | --- | --- | --- | --- | --- |
| Number of patients at risk (Platinum sensitive ) | 79 | 39 | 14 | 2 | 0 |
| Number of patients at risk (Platinum resistant) | 40 | 15 | 7 | 2 | 0 |
| Months | 0 | 6 | 12 | 18 | 24 |

| **Table 8:** Number of patients at risk (censored) during specific time intervals (months) for median progression-free survival (PFS) among Line of treatment subgroups. | | | | | |
| --- | --- | --- | --- | --- | --- |
| Number of patients at risk (First line) | 87 | 44 | 15 | 2 | 0 |
| Number of patients at risk (Second line) | 32 | 10 | 5 | 2 | 0 |
| Months | 0 | 6 | 12 | 18 | 24 |
